# Warnings to Counter Choice Blindness for Identification Decisions: Warnings Offer an Advantage in Time but Not in Rate of Detection

**DOI:** 10.3389/fpsyg.2018.00981

**Published:** 2018-06-13

**Authors:** Anna Sagana, Melanie Sauerland, Harald Merckelbach

**Affiliations:** Section Forensic Psychology, Department of Clinical Psychological Science, Faculty of Psychology and Neuroscience, Maastricht University, Maastricht, Netherlands

**Keywords:** pre-warning, post-warning, enlightening, choice blindness, misinformation

## Abstract

Choice blindness for identification decisions refers to the inability of eyewitnesses to detect that an originally recognized target was swapped for a non-identified lineup member. The robustness of the effect calls for measures that can prevent or reduce the negative consequences of choice blindness manipulations. Here, we investigated whether pre- and post-warnings given to participants about the possibility of mistakes reduces choice blindness for identification decisions. Participants (*N* = 119) were presented with identifications they never made and were asked to justify those decisions. Either before or after the presentation of the manipulated identification outcome, participants were or were not warned about the possibility of mistakes in the identification process. Although warnings were not sufficient to reduce choice blindness for identification decisions they provided a time-related detection advantage. Pre-warned participants questioned the legitimacy of the manipulated outcome sooner (i.e., concurrent detection) than participants in other conditions. Hence, pre-warnings can help detect mistakes in the identification procedure at an earlier stage, before they contaminate the memory of the witness and other pieces of evidence. From a theoretical stance, our findings attest to the strength of self-suggestion and indicate that choice blindness effects are deeply rooted in cognition.

## Introduction

Choice blindness refers to a difficulty to detect discrepancies between a choice and its outcome and a tendency to justify choices which were never made (Johansson et al., 2005). In the classic choice blindness paradigm, participants make a binary decision and are subsequently presented with the opposite of their choice. At that point, they are asked to explain the reasons behind their supposed choice. To establish whether they detected the manipulation, participants have the opportunity to communicate their concerns at two points in time. The first is immediately after the presentation of the manipulation when asked to justify their choice (i.e., concurrently). The second is at the end of the experiment by means of specific questions that point to the possibility of a manipulation (i.e., retrospectively). Typically, the overwhelming majority (>70%) of participants fails to detect the manipulation of their decision and endorses the manipulated outcome.

Previous research has largely concentrated on choice blindness for eyewitness identification decisions (Sagana et al., 2013, 2014b, 2015). In one of the early demonstrations, Sagana et al. (2014b) had participants watch a number of mock crime films and then identify the targets from simultaneous target-present lineups. Following each forced-choice recognition decision, participants were confronted with the identified face and were asked to provide reasons for their decision. On some of the trials participants were confronted with an originally non-chosen face from the lineup. After an ecologically valid 48 h interval between the identification and the confrontation with the manipulated outcome, participants detected merely 32% of the manipulations concurrently and 29% retrospectively (Experiment 3). Hence, by the end of that study, only 61% of the manipulations had been detected. The effect of choice blindness for identification decision has been replicated under naturalistic encoding conditions, employing a field study methodology, target-present as well as target-absent lineups, and a less memory taxing and cognitively demanding task (i.e., two rather than 16 identifications; Sagana et al., 2013, 2015). More recent experiments have shown that choice blindness manipulations can also have long-lasting effects. Cochran et al. (2016) found that eyewitnesses who fail to detect the manipulation are more likely to change their identification decision in the direction of the manipulation when asked to perform the identification task a second time.

The practical importance of these findings is immense. In the short-run, choice blindness manipulations can lead to incrimination of innocents and impede the investigation of a crime. In fact, we know that such manipulations have occurred in real cases (see case of Bernard Maughan; Wolchover, n.d.) and that procedural errors (Saks and Koehler, 2005; Kassin et al., 2013) and police misconduct (Ridolfi and Possley, 2010) are among the leading causes of wrongful convictions. In the long-run, given the lasting effects of choice blindness manipulations on eyewitness memory and decision making, witnesses can no longer provide accurate information to the police and the court. Therefore, prevention or reduction of the effects of choice blindness manipulations on identification decisions forms an important issue.

There are reasons to believe that warning witnesses about the possibility of errors could be effective in reducing choice blindness for identification decisions. Earlier choice blindness experiments indicated that, at times, the detection of one manipulation increases the odds of detecting subsequent manipulations (e.g., Johansson et al., 2005; Sauerland et al., 2013). A reason for this cascading effect could be that the detection of the first manipulation serves to caution the participant that mistakes of this sort are possible. Hence, participants might monitor the outcome of the subsequent choices more carefully and are therefore more likely to detect future manipulations. Furthermore, an early warning about the possibility of mistakes may offer participants a rational explanation if they experience dissonance during the confrontation with a manipulated outcome; increasing the report rate at the end of the study. Providing a reasonable alternative explanation has been successful in reducing the belief in false information (Johnson and Seifert, 1994; Tenney et al., 2009).

Additionally, warnings have been used widely, and mostly successfully, to reduce the negative effects of misleading post-event information (for reviews see Loftus, 2005; Oeberst and Blank, 2012). For example pre-warning participants about the likely presence of misleading information helps to resist misinformation and its damaging effects (e.g., Underwood and Pezdek, 1998; Chambers and Zaragoza, 2001), possibly because pre-warnings enhance participants’ attention to the task and help them scrutinize post-event information for discrepancies (Greene et al., 1982). A recent meta-analysis suggests that post-warnings are also effective as they can reduce the misinformation effect to less than half its normal size (Blank and Launay, 2014). However, the degree to which the warning specifies the incident or the source of the misinformation is a significant moderator, with more precise warnings leading to greater reductions in misinformation endorsement. Moreover, warnings that specify not only the presence but also the logic behind the misinformation, a procedure known as enlightenment (c.f., Oeberst and Blank, 2012), have greater potential in reducing the misinformation endorsement than simple post-warnings. That is because participants are assisted in their search for and discrimination between two candidate answers (Oeberst and Blank, 2012).

To examine this issue in a systematic fashion, our study tested whether warning participants about the possibility of mistakes could prevent or reduce the negative influence of choice blindness manipulations in an eyewitness setting. To this end, we investigated the potential reduction in blindness rates as a result of a simple pre-warning, a simple post-warning, and an enlightening warning. The pre- and the post-warning cautioned participants about the possibility of an error in the identification procedure. These warnings were purposefully kept generic to mimic conditions where investigators are unaware of the source of the problem but they nonetheless use warnings as a protective measure. The enlightening warning was a more specific warning. Therein, we explained the logic behind the error (i.e., that the original choice might get lost due to procedural errors) in an attempt to assist participants to discriminate between the original choice and the source of the error (i.e., manipulation). First, we expected that pre- and post-warned participants would exhibit higher detection rates than non-warned participants (control group, H1). Second, we hypothesized that the pre-warning would result in higher detection rates compared with the other warning conditions (H2). Thirdly, because the enlightening warning is more elaborate than the simple post-warning, participants who were enlightened were expected to display higher retrospective and overall detection rates than participants who received a simple post-warning (H3).

Aside from the effect of warnings, the current study also expands on the role of identification accuracy, lineup selection, and post-identification confidence. Earlier investigations revealed an inconsistent relationship between identification accuracy and choice blindness for identification decisions. While some studies showed no meaningful effect of accuracy on detection rates (Sagana et al., 2014a; Stille et al., 2017), other studies reported a positive (Sagana et al., 2013, 2014b) or a negative (Sagana et al., 2015) relationship. From a theoretical standpoint, the role of accuracy is equally puzzling. Lacking a strong memory trace for the target (i.e., low identification accuracy) means that an effective comparison between the original target and the presented outcome is unfeasible. From that point of view, choice blindness is the result of memory decay. However, we know that choice blindness occurs also for tasks that are not memory taxing (Johansson et al., 2005; Hall et al., 2010). Findings like these suggest that poor memory is not a necessary prerequisite for choice blindness and that another, yet to be discovered, mechanism is at play. Here, we explored whether identification accuracy moderates detection rates without prior expectations regarding the direction of the effect.

Furthermore, we expected lineup rejections that were turned into selections (i.e., choice reversals) to be more readily detected than selections in which the outcome was exchanged with a previously unidentified lineup member (i.e., choice exchange, H4) as earlier demonstrated by Sagana et al. (2015; blindness rate: reversal 12–18%, exchange 71–82%). The authors argued that to detect a manipulation in the case of choice exchanges, one needs to concentrate on the characteristics of the manipulated face and the attention needs to be drawn to the specifics of the decision (Sagana et al., 2015). However, to detect a manipulation in the case of choice reversal, one’s attention needs to be drawn simply to the presence of a face where there should have been none. Therefore, a choice reversal is a distinct category conversion that directly contradicts the original decision and is, thus, more readily detected than a choice exchange. The greater attentional shift for between-category changes, such as choice reversals, than for within-category changes, such as choice exchanges has also been demonstrated for the related phenomenon of change blindness (Lyyra et al., 2014). This is in line with Sauerland et al. (2015) who altered the reported frequency with which participants had performed certain transgressions. Detection rates were more pronounced when participants had indicated never committing a certain transgression in the past (no transgression history) and this indication was changed to 3 times (e.g., changing from *I have never stolen a bike* to *I have stolen a bike 3 times*), than for alterations of frequencies that were larger than 0 (e.g., *I have stolen a bike 3 times* to *I have stolen a bike 6 times*). The latter manipulation only entails a change within level of transgression history, whereas the former entails a switch from no transgression history (*have never done this before*) to transgression history (*have done this before*). The findings were explained in light of the source monitoring framework (Johnson et al., 1981; Johnson et al., 1993). According to this framework, we use the feeling of familiarity associated with an event as an indicator of its actual occurrence (Johnson et al., 1993; Polage, 2012). Alterations that cause discrepancies in participants’ feeling of familiarity, such as changing a no transgression history response to a transgression history response, should be detected with more ease than alterations that do not cause such discrepancies, changing one history response to a different history response (Sauerland et al., 2015).

Finally, regarding post-identification confidence we explored the possibility that the acceptance of choice blindness manipulations would be associated with identification decisions that were made with a lower degree of confidence. People who (are led to) distrust their memories tend to rely on external sources and cues for validation (Gudjonsson, 2003). As such, they are more likely to accept misinformation (Van Bergen et al., 2010). Accordingly, participants who are not confident in their identification decisions – that is, participants who display low trust in their memory – should be more willing to accept a manipulated outcome than those who are confident. Previous work suggested that indeed low confidence may facilitate the acceptance of the manipulated outcome (Sagana et al., 2013). Therefore, we expected that when identification decisions were made with lower rather than a higher degree of confidence participants would be more likely to endorse the manipulated outcome (H5). Additionally, we were interested in examining whether the mere presence of choice blindness manipulations would undermine the confidence assigned to one’s identification decision at a later point in time as shown in earlier studies (H6; Sagana et al., 2013). We expect that the manipulation would contaminate participants’ estimates of confidence in a similar way to (negative) post-identification feedback (Wells and Bradfield, 1998; Douglass and Steblay, 2006; Steblay et al., 2014). Participants might experience a discrepancy between the original choice and the manipulated outcome at a metacognitive level. Specifically, when presented with the manipulated outcome participants’ ecphoric experience (i.e., the subjective sense of similarity between a stimulus and a person’s memory, (Tulving, 1981), should be weak because the manipulated outcome does not accord with the memory for the selected face. Given that witnesses use ecphoric experiences as a basis for their confidence judgments (Sporer et al., 1995; Wells et al., 2003; Charman and Wells, 2012), we expected that their confidence estimated after the presentation of the manipulated outcome will be lower compared to before the presentation of the manipulated outcome. Such a development could have serious consequences in legal settings as low confidence identifications are met with skepticism in court.

## Materials and Methods

### Participants

In total 128 participants took part in the study. We had to exclude six participants because we could not verify their detection rate, two participants because they recognized someone from the lineups and one participant because of a missing informed consent. This left data from *N* = 119 participants (*M*_age_ = 20.4, *SD*_age_ = 2.2, *age range*: 18–26) for analysis. Participants were mostly psychology (86.6%), medicine (6.8%), and law students (1.7%). Participation was voluntary and participants received course credit in return for their participation. All participants were naïve to the actual purpose of the study and were tested individually. The study was approved by the standing Ethical Committee of the faculty and written informed consent was gained from all participants.

### Design

**FIGURE 1:**
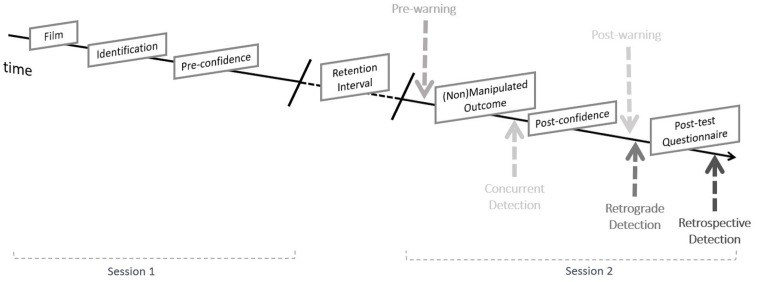
Overview of the experimental procedure along with the point in time detections were recorded. Participants received only one warning (pre or post). Detections rates were measured at three points in time across all participants.

For this study we employed a mixed 4 (warning condition: pre-warning vs. simple post-warning vs. enlightening vs. no-warning; between-subjects) × 2 (lineup mode: target-present vs. target-absent; within-subjects) factorial design. Based on the identification outcome two additional variables were derived, namely lineup selection (selection vs. rejection) and identification accuracy (accurate vs. inaccurate). Warning condition, lineup selection, and identification accuracy served as predictors of detection in all ensuing analyses. The dependent variables were detection (H1–4) and confidence (H5–6). Detection rates were measured three times: concurrently, retrograde, and in retrospect. **Figure 1** displays the point within the procedure each detection rate was recorded. Concurrent detection refers to the spontaneous detection of a manipulation when it takes place and was therefore coded when participants reported that they noticed the manipulation immediately after being confronted with the manipulated outcome. Retrograde detection refers to the spontaneous detection of a manipulation that it takes place after participants are asked to reflect upon the task. It was thus coded when detection occurred at the first half of the post-test questionnaire (see section “Post-test Questionnaire”); when asked whether they had noticed anything strange during the experiment. Retrospective detection refers to the detection that takes place after participants are notified about the possibility of manipulations. Therefore, it was coded when participants indicated that they belonged to the experimental condition at the second half of the post-test questionnaire and correctly specified the manipulated trial(s) (see section “Post-test Questionnaire”). The sum of all three types of detections constitutes the overall detection rate. Confidence was measured at two points in time; immediately following the identification decision, but prior to the presentation of the manipulation (pre-manipulation confidence) and following the presentation of the manipulated decision (post-manipulation confidence).

### Materials

#### Stimulus Films

Stimulus films were taken from Sagana et al. (2014b). In brief, four films showing minor offenses were used as stimuli (*M*_duration_ = 178 s, range: 160–214 s). Each film involved four different actors (perpetrator, victim, and two bystanders). All targets within each film were shown from frontal and side views for a minimum of 76 s, with close-ups of 2–9 s. The films were presented in counterbalanced order. With the exception of retrospective detection for one film, *x^2^*(1, 119) = 8.59 *p* = 0.004, *phi* = 0.27, the order of the films did not affect detection rates (*x^2^*s ≤ 1.88, *p*s ≥ 0.199, *phi*s ≤ 0.13) or identification accuracy (*x^2^*s ≤ 4.22, *p*s ≥ 0.054, *phi*s ≤ 0.19). Given that (a) there is no previous record of a similar film effect, (b) the effect is small in magnitude, and (c) it is very specific (one film in only one detection category), we will not discuss this factor any further. A detailed description of the films can be found in Sagana et al. (2014b).

#### Lineups

We created 24 simultaneous photo lineups. For the four perpetrators and the four victims, both target-present and target-absent lineups were created. For the bystanders only target-present lineups were constructed. However, we were only interested in the four perpetrator identifications, which bear the strongest relevance for criminal investigations. Thus, neither the victim nor the bystander lineups were analyzed. The victim and bystander lineups were used to avoid participants from getting suspicious about the purpose of the study. Additionally, the target-absent victim lineups served to reduce the uneven base rate of target-present and target-absent lineups, which might have had increased the tendency of participants to choose. To avoid anchoring effects the target-present and target-absent lineups were counterbalanced within and across films and across perpetrator and victim targets. Specifically, for two films the perpetrator lineups were target-absent and the victim lineups were target-present, whereas the reverse was true for the other two films.

Head and shoulder photos were selected to match the description of the respective targets (effective lineup sizes determined as Tredoux’s Es ranged from 2.50 to 5.76; Tredoux, 1998, 1999). Each lineup included one target or replacement of the target, and five distractors. The pictures were presented on a 2 × 3 array and the size of the photos, as presented on the computer screen, was 4 cm × 5 cm. On the right side of the screen, next to the six photos, participants were given the option to reject the lineup. Participants had unlimited time to make their choice and they were instructed that the target may or may not be present in the lineup.

#### Warnings

We employed one pre-warning and two post-warning (simple post-warning and enlightening) conditions.

##### Pre-warning

Participants in the pre-warning condition were informed about the possibility of procedural errors before being confronted with the manipulated identification outcome(s). Participants received the following instruction:

“Your task today is to explain the reasons for your identification decision to the judge. Keep in mind that procedural errors can occur in eyewitness identification procedures!”

##### Simple post-warning

In this condition, participants were informed about the possibility of procedural errors right after they had been exposed to the manipulated identification outcomes. The content of this warning was identical to that of the pre-warning.

##### Enlightening

Participants in this condition, like those in the simple post-warning condition, were informed about the possibility of procedural errors shortly after they had been exposed to the manipulated identification outcomes. However, here participants received a warning that aimed at explaining the logic behind the manipulation and separating the memory source of the original choice from that of the manipulated identification decision. Participants received the following instruction:

*“*It is very important for this experiment that you answer the following questions properly. This experiment is actually about the psychology of eyewitness memory and the influence of discrepant post-identification information. Imagine you witnessed a theft and that you have identified the thief from a series of photos. Later, you are asked to testify in support of your identification in court. However, due to a procedural error, a person different to the one you identified has been put on trial. Take this scenario into account when answering the following questions and rely exclusively on your own memory of the perpetrators!*”*

#### Post-test Questionnaire

Participants were given a post-test questionnaire to determine whether they had noticed the manipulation but refrained from revealing it. In the first half of this questionnaire, participants were asked whether they had noticed anything strange during the experiment. If they responded affirmatively, they were invited to provide details. In the second half of the questionnaire, participants were *misleadingly* informed that the present study employed two experimental conditions, one in which some of the choices were manipulated (i.e., “Some of the perpetrators’ faces that were presented to you today were not the ones that you originally identified during Session 1”; as in the actual experiment), and one control condition where this was not the case. Participants had to indicate to which condition they believed they had been assigned to. If a participant indicated that s/he belonged to the manipulated condition, s/he also had to indicate how many times s/he had noticed a manipulation and had to indicate the specific targets (for similar procedure see e.g., Johansson et al., 2008; Hall et al., 2010; Sagana et al., 2013, 2014b, 2015; Sauerland et al., 2013).

### Procedure

The procedure was analogous to that of Sagana et al. (2014b; Experiment 3) with the key difference that, depending on the experimental condition, participants were either pre-warned, post-warned, or not warned and that they received both target present and target-absent lineups. **Figure 1** provides an overview of the procedure along with the point in time detections were recorded. Participants were informed that we were interested in eyewitness testimony but were naïve to the actual purpose of the study.

The experiment involved two sessions. In session 1, participants first watched a stimulus film and immediately after its end they were asked to identify all four actors from separate simultaneous photo lineups. Participants were instructed that the target may or may not be present and were given the option to reject the lineup. Following their identification decision participants were asked to rate how confident they were in their decision (i.e., pre-manipulation confidence) on an 11-point Likert scale. The same procedure was followed for all films. When participants had finished with the identifications for all four films, session 1 was terminated.

Participants returned to the lab 48 h later and were asked to imagine they were in the courtroom and they had to explain their identification decisions to the judge. In addition to these instructions participants in the pre-warning condition were presented with the warning, which they read in the presence of the experimenter. Participants were presented with the face of the identified suspect and wrote down their reasons for that choice on paper sheets provided. If participants had rejected the lineup, they were presented with a pencil drawing of a “faceless” face and were asked to explain why they had rejected the lineup. The same procedure was followed for all four films. However, for two of the four suspects, participants were presented with a lineup member different from the one they had originally chosen. If participants had initially rejected the lineup, they were presented with the photo of the perpetrator in the target-present condition and the photo of the replacement in the target-absent condition, instead of the sketch photo. Immediately after the presentation of the (manipulated) identification decision participants were asked to rate how confident they now felt about their decision (post-manipulation confidence) on an 11-point Likert scale. Once participants had finished this task for all four films, those belonging in the post-warning and the enlightening condition received the respective warning. The warning was read in the presence of the experimenter. Then, all participants filled in the post-test questionnaire. Finally, participants were thanked and fully debriefed.

## Results

### Choice Blindness

**FIGURE 2:**
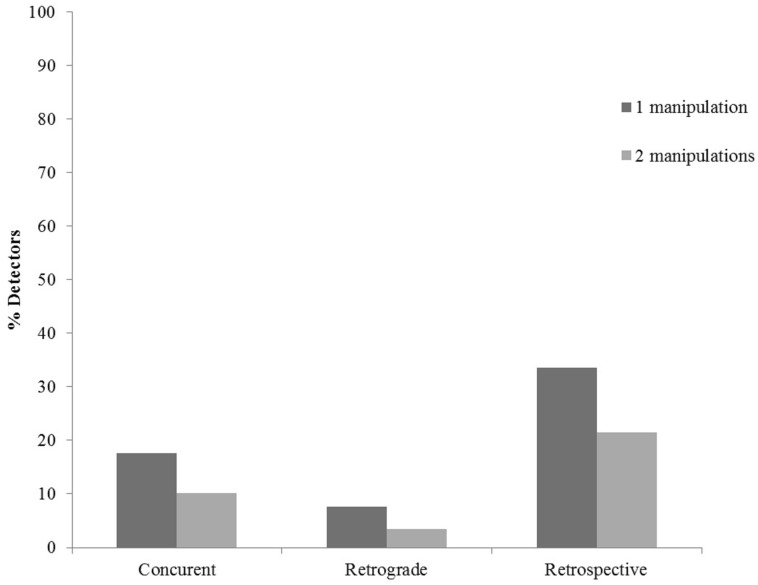
Proportion of participants who detected one or two manipulations concurrently, retrogradely, and in retrospect. The data are aggregated across four warning conditions.

At a participant level and across all conditions and detection categories, 35.0% of the participants detected both manipulations, 58.8% detected one of the two manipulations, while 6.2% detected none of the manipulations. **Figure 2** presents the distribution of concurrent, retrograde, and retrospective detections at the participant level.

In total, 119 × 2 = 238 manipulations were performed, thus 238 detections were possible. At the trial level and across all warning conditions 18.9% of the manipulations were detected concurrently, an additional 5.5% were detected retrogradely and 36.6% in retrospect. Accordingly, 60.9% of the manipulated trials were detected at some point in time. This rate is as a conceptual replication of overall detection rate reported in Sagana et al. (2014b) and fits well within the detection rate for identification decisions (Sagana et al., 2013, 2015; Cochran et al., 2016).

### Detection Rate as a Function of Warnings

**FIGURE 3:**
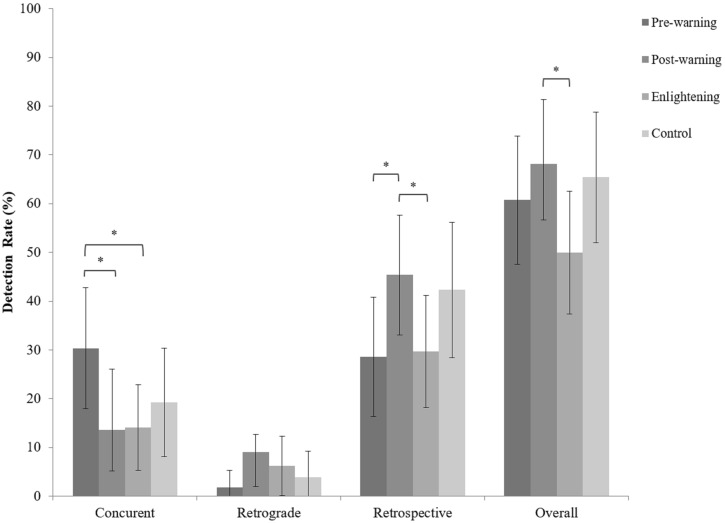
Proportion of concurrent, retrograde, and retrospective detection rate as a function of warning conditions. Error bars represent bootstrapped 95% confidence intervals. Asterisk represents a statistically significant difference (^∗^*p* < 0.05).

To investigate the effect of warnings on detection rates we applied a regression methodology known as generalized estimating equations (GEE) analysis. The method enables the incorporation of the repeated observations for all the perpetrator lineups as it accounts for the correlated residuals via the specification of a working correlation matrix (Hanley et al., 2003). Concurrent, retrospective, and overall detection rates were the dependent variables with warning condition as predictor. Given the small amount of retrograde detections (13 trials, 5.5%) no statistical analyses could be performed for this type of detection. It is of note, however, that the majority of retrograde detections were observed in the two post-warning conditions (**Figure 3**). It seems that the presence of a warning triggers participants to voice their concerns about the manipulations.

First, we examined the hypothesis that warnings would increase detection rates (H1). We therefore performed GEE analysis with detection rates as outcome variable and the warning condition as predictor. The control condition served as the base in these comparisons. The Wald criterion demonstrated that the predictor warning condition did not make a significant contribution to predicting any type of detection, all Wald χ^2^s (3, 238) ≤ 6.69, *p*s ≥ 0.082. With the exception of concurrent detection for the pre-warned participants (1.83), the magnitude of the odd ratios (ORs) spanned from no effect (0.53) to small effects (1.13) (Cohen, 1988; Chen et al., 2010) supporting the idea of a lack of association between the warning conditions and the detection rates. These findings are not in line with our hypothesis. The inferential statistics for this analysis can be found in **Table 1**.

**Table 1:** Summary of GEE analysis for concurrent, retrospective and overall detection rates as a function of warning condition.

	*OR*	*95% CI*	*SE*	*Wald x^2^*	*p*
**Concurrent detection**					
Warning condition (Base = Control)					
Pre-warning	1.83	0.75–4.48	0.46	1.76	0.185
Post-warning	0.66	0.25–1.77	0.50	0.67	0.414
Enlightening	0.69	0.26–1.84	0.50	0.56	0.456
Intercept	0.24	0.12–0.47	0.35	16.63	<0.001
**Retrospective detection**					
Warning condition (Base = Control)					
Pre-warning	0.54	0.24–1.21	0.41	2.21	0.137
Post-warning	1.13	0.55–2.36	0.37	0.18	0.733
Enlightening	0.58	0.27–1.24	0.39	1.98	0.159
Intercept	0.73	0.42–1.27	0.28	1.22	0.269
**Overall detection**					
Warning condition (Base = Control)					
Pre-warning	0.82	0.37–1.79	0.40	0.25	0.616
Post-warning	1.13	0.52–2.45	0.39	0.10	0.748
Enlightening	0.53	0.25–1.12	0.38	2.74	0.098
Intercept	1.89	1.07–3.34	0.29	4.76	0.029

*Warning condition predictors coded as 0 for control, 1 for pre-warning, 2 for post-warning, and 3 for enlightening. CI, confidence interval; SE, standard error; OR, odds ratio.*

Next, we examined the hypothesis that pre-warning will be advantageous for detection rates compared with other warning conditions (H2). *Post hoc* comparisons with the pre-warning condition as base revealed that for concurrent detection, detection rate was higher for the pre-warning than the post-warning, Wald χ^2^(1, 122) = 4.83, *p* = 0.028, *OR* = 2.76 and the enlightening condition, Wald χ^2^(1, 120) = 4.49, *p* = 0.034, *OR* = 2.66. These findings are in line with our hypothesis. **Figure 3** displays mean detection rates across all conditions. For retrospective and overall detection, there were no significant differences between the pre-warning and the two post-warning conditions, all Wald χ^2^s(1, 120–122) ≤ 0.138, *p*s ≥ 0.240, *OR*s ≤ 1.54. The only exception was the marginally lower retrospective detection rate for the pre-warning than the post-warning condition, Wald χ^2^(1, 122) = 3.85, *p* = 0.057, *OR*s = 0.48. However, here too the effect size is very small. Taken together, these results imply that the advantage of a pre-warning is in the timing of the detection. Although the overall detection rates were not increased, the detection of the manipulation is likely to take place earlier in time (i.e., concurrently) if participants are pre-warned.

Finally, we turned to the hypothesis that the enlightening warning would be more effective than a simple post-warning (H3). This comparison is meaningful only for detection rates following the administration of these warnings; thus only for retrospective and overall detection. Comparisons suggest that this was not the case. For retrospective detection, we observed no differences between the two conditions (**Figure 3**), Wald χ^2^(1, 130) = 3.40, *p* = 0.065, *OR* = 1.97, and the pattern of detection was opposite to our expectations. Likewise, the overall detection rate for the simple post-warning condition was significantly higher than that of the enlightening condition, Wald χ^2^(1, 130) = 4.36, *p* = 0.032, *OR* = 2.15. This finding is contrary to our hypothesis. Interestingly, the odd ratios for these effects were medium suggesting that the opposite of our hypothesis might be true.

### Detection Rate as a Function of Identification Accuracy and Lineup Outcome

**FIGURE 4:**
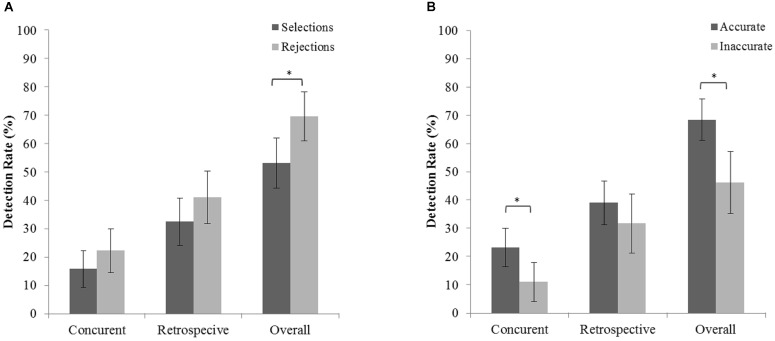
Proportion of concurrent, retrospective, and overall detection rate as a function of lineup selection **(A)** and identification accuracy **(B)**. Error bars represent bootstrapped 95% confidence intervals. Asterisk represents a statistically significant difference (^∗^*p* < 0.05).

Having established the role of warnings on choice blindness for identification decision we turned to the effect of identification accuracy (accurate vs. inaccurate) and lineup selection (selecting vs. rejecting) on detection rates. Participants selected someone from the lineup in 52.9% of the cases and were accurate in 65.5% of the cases. In the initial analyses, we included both main effects and the two-way interaction in the equation. Next, we sequentially excluded non-significant effects. **Table 2** shows the final models (based on the QICC independence model criterion) for three types of detection. Identification accuracy, but not lineup selection significantly contributed to predicting concurrent detection (final model fit QICC = 229.28). Specifically, manipulations following accurate identifications were 1.5 times more likely to be concurrently detected than manipulations following inaccurate identifications (**Figure 4**). For retrospective detection, the final model had no predictive value (final model fit QICC = 314.65). For overall detection, accuracy and lineup selection (but not the interaction) were significant predictors of detection (final model fit QICC = 307.92). Overall, manipulations following accurate identifications were 2.4 times more likely to be detected than those following inaccurate identifications (**Figure 4**). This finding is in line with previous investigations (Sagana et al., 2013). Furthermore, manipulations following a lineup rejection were 1.9 times more likely to be detected than manipulations following a lineup selection. This finding is in accordance with our hypothesis (H4) and earlier findings (Sagana et al., 2015; Sauerland et al., 2015).

**Table 2:** Summary of GEE analysis for concurrent, retrospective, and overall detection rates identification accuracy and lineup selection.

	*OR*	*95% CI*	*SE*	*Wald x^2^*	*p*
**Concurrent detection**					
Identification accuracy	2.43	1.11–5.34	0.40	4.91	0.027
Intercept	3.33	2.29–4.83	0.19	40.14	<0.001
**Retrospective detection**					
Lineup selection	1.44	0.85–2.45	0.19	1.85	0.060
Intercept	1.43	0.98–2.09	0.27	3.53	0.173
**Overall detection**					
Lineup selection	1.91	1.11–3.29	0.28	5.39	0.020
Identification accuracy	2.42	1.38–4.21	0.28	9.68	0.002
Intercept	0.33	0.21–0.52	0.23	22.82	<0.001

*Accuracy coded as 0 for inaccurate and 1 for accurate. Lineup selection coded as 0 for rejection and 1 for selection. CI, confidence interval; SE, standard error; OR, odds ratio.*

### The Role of Confidence in Relation to Detection Rates

To test the hypothesis that detection was associated with higher post-identification confidence than blindness (H5), we performed GEE analyses with each detection type (concurrent, retrospective, and overall) as dependent variable and pre-manipulation confidence as predictor. The results were parallel for concurrent, retrospective, and overall detection. Post-identification confidence was not a significant predictor of detection, all Wald χ^2^s(1, *n* = 238) ≤ 0.70, *p*s *≥* 0.485, *OR*s ≤ 1.04. The results do not support our hypothesis.

**FIGURE 5:**
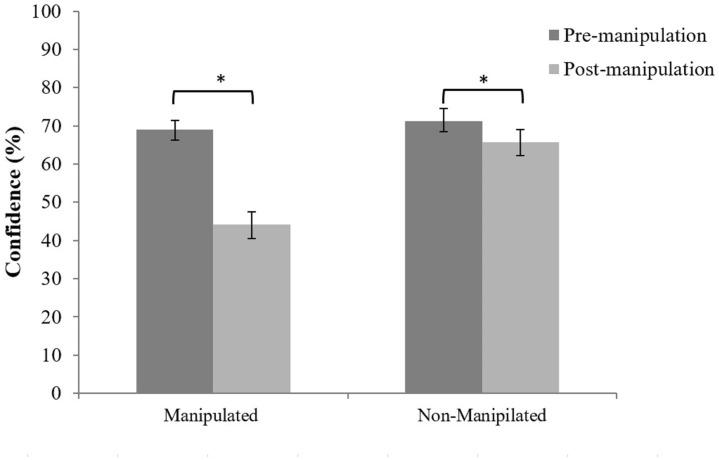
Identification confidence rates before (pre-manipulation) and after (post-manipulation) the presentation of the manipulated outcome and as a function of manipulation status. Error bars represent bootstrapped 95% confidence intervals. Asterisk represents a statistically significant difference (^∗^*p* < 0.05).

Then, we turned to the possibility that choice blindness manipulations affect the confidence in one’s identification decision (H6). To that end we performed a repeated measures ANOVA with confidence (pre- vs. post-manipulation) as dependent variable and manipulation status (manipulated vs. non-manipulated lineups) and the order of the lineup (first vs. second lineup) as independent variables. The inclusion of the non-manipulated lineups in this analysis is necessary to verify that any observed differences are not a mere effect of time lapse. The three-way interaction of time, manipulation status and lineup order was not significant, *F*(1,118) = 0.06, *p* = 0.810, $\eta_{p}^{2}$ = 0.001. Neither was the two way interaction between time and lineup order *F*(1,118) = 0.88, *p* = 0.349, $\eta_{p}^{2}$ = 0.008, or between manipulation status and lineup order, *F*(1,118) = 0.16, *p* = 0.900, $\eta_{p}^{2}$ = 0.001. Finally, there was no significant main effect of lineup order, *F*(1,118) = 0.12, *p* = 0.727, $\eta_{p}^{2}$ = 0.001. However, the interaction term of time and manipulation status was significant, *F*(1,118) = 51.03, *p* < 0.001, $\eta_{p}^{2}$ = 0.306, as were the main effects of time, all *F*(1,118) = 98.01, *p* < 0.001, $\eta_{p}^{2}$ = 0.458, and manipulation status, *F*(1,118) = 69.43, *p* < 0.001, $\eta_{p}^{2}$ = 0.374. After splitting the interaction term by manipulation status, the analyses revealed that for the manipulated lineups, there was a significant drop in confidence from before (pre-manipulation = 69.0%, 95% CI [66.3, 71.5]) to after the presentation of the manipulation (post-manipulation = 44.1%, 95% CI [40.5, 47.5]), *F*(1,118) = 63.84, *p* < 0.001, $\eta_{p}^{2}$ = 0.908. Although this was the case for non-manipulated trials as well, all *F*(1,118) = 5.74, *p* = 0.016, $\eta_{p}^{2}$ = 0.047, here the drop of confidence from before (pre-manipulation = 71.2%, 95% CI [68.4, 73.7]) to after the manipulation (post-manipulation = 65.8%, 95% CI [62.3, 69.1]) was not as dramatic as that for the manipulated trials, as evidenced by the different magnitude of the effect sizes (**Figure 5**). Hence, it seems that the mere presence of a manipulation is sufficient to lower the confidence in one’s eyewitness identification decision.

## Discussion

The present study investigated whether warning witnesses about the possibility of procedural mistakes can reduce choice blindness for eyewitness identification decisions. We believed that warnings would increase participants’ attention (e.g., Blank and Launay, 2014) and induce a temporary state of skepticism, which may maximize the ability to discriminate between true and false information (Lewandowsky et al., 2012). Furthermore, warnings should give participants a reasonable, alternative explanation (e.g., a mistake) that could help them “fill the gap” in the presence of a changed outcome (Johnson and Seifert, 1994; Tenney et al., 2009). Contrary to expectations, we did not find a meaningful overall increase in detection rates because of warnings. The magnitude ORs was small and the 95% confidence intervals indicated a lack of an association between detection and warning condition. Despite the lack of a detection advantage, pre-warnings elicited a time-related advantage in detection. Pre-warned participants raised their concerns about the legitimacy of the outcome sooner (i.e., concurrent detection) than participants in other conditions. This finding is of practical importance as it demonstrates that pre-warnings can help detect and correct procedural mistakes sooner; that is, before they can contaminate other pieces of evidence.

The observation that warnings are inapt to counter blindness manipulations supports the idea that choice blindness is not the result of deliberate attentional underperformance or social influences (e.g., Johansson et al., 2008; Merckelbach et al., 2011; Sagana and Sauerland, 2017). Rather, these findings attest to the strength of self-suggestion (Stille et al., 2017). In our and similar studies (e.g., Johansson et al., 2005; Cochran et al., 2016), participants perceive the manipulated outcome as their own choice. Past research has shown that people see themselves in a more positive light than others do (Mabe and West, 1982; Harris and Schaubroeck, 1988) and tend to be less critical of their own than other people’s arguments (Trouche et al., 2016). Additionally, more trusting sources, as one’s self usually is, lead to higher endorsement of post-event and/or misleading suggestions (e.g., Hope et al., 2008; Skagerberg and Wright, 2009). Therefore, it is not odd that choice blindness for one’s identification decisions could not be trumped by warnings.

In striking contrast to studies on enlightening and misinformation (Oeberst and Blank, 2012), participants in the enlightening condition did not benefit from the elaborate warning. We believe that the ineffectiveness of the enlightening procedure in the present context could be due to the post-test questionnaire (filled in by all participants) may have constituted an even stronger and more specific cautionary notice than our warnings. It has consistently been proposed that for warnings to be effective they need to be specific and clearly explain the ongoing effects, rather than generally mention that misinformation may be present (e.g., Ecker et al., 2010; Oeberst and Blank, 2012). The instructions in the post-test questionnaire may thus have been the admonition that resulted in the utmost increase in detection rates. This could also explain the lack of differences between the control and the simple post-warning condition. Hence, it could be argued that a more specific (pre- or post) warning could improve detection rates. While this is possible, a warning that explicitly cautions the participant that changes have occurred would lack ecological validity. That is because in real cases one cannot know if and what kind of changes occurred. The observation about the role of the post-test questionnaire, however, suggests that typically reported detection rates may already reflect participants’ maximum detection capacity. Therefore, in the absence of any cautionary notice, detection rates might be considerably lower, approximating concurrent detection rates. This observation that participants at best detect only half of the manipulations implies that choice blindness for one’s (identification) decisions stems from fundamental limitations in perception and cognition; potentially stemming from a need for consistency and relative stability in the external world.

Apart from the role of warnings on choice blindness for identification decisions, we also investigated the role of identification accuracy, lineup selection, and post-identification confidence. Identification accuracy was a predictor of detection rates. Consistent with earlier findings, accurate identification decisions were associated with higher concurrent (Sagana et al., 2014b) and overall detection rates (Sagana et al., 2013). It seems that weak memory, as illustrated in low accuracy rates, makes witnesses susceptible to choice blindness manipulations, possibly because they may lack the means for an effective comparison between the original target and the presented outcome. To further explore the role of identification accuracy in choice blindness manipulations future research should consider disentangling remember from know identification decisions (for similar approach see Sauerland and Sporer, 2009; Palmer et al., 2010), as the two decision types are theorized to be indicative of different degrees of memory strength (for a discussion see Wixted, 2009). However, this finding should not be interpreted as evidence that weak memory is the reason underlying blindness. If memory strength had a causal relationship with choice blindness one would expect a persistent pattern of findings across studies. However, identification accuracy is not a stable predictor of detection. While some studies showed a positive effect of accuracy on detection rates (Sagana et al., 2013, 2014b) others report no meaningful (Sagana et al., 2014a; Stille et al., 2017) or even a negative (Sagana et al., 2015) relationship. The unstable nature of the effect could also reflect the fact that an identification decision is not just the product of one’s memory. Instead, identification decisions depend heavily on one’s response criterion (e.g., Wells, 1984; Lindsay and Wells, 1985), perceptions about the task (e.g., Brewer and Wells, 2006) and metacognitive influences (e.g., Johnson et al., 1993; Horry et al., 2016). Importantly, if memory strength was the reason underlying choice blindness, then blindness should dissipate under conditions of strong memory. However, choice blindness can arise for decisions that are not memory taxing (e.g., Johansson et al., 2005; Hall et al., 2010), thus memory decay is a sufficient but not a necessary condition for choice blindness.

With respect to lineup selection, we found that when lineup rejections were turned into positive identifications (i.e., choice reversals) participants were 1.9 times more likely to detect the manipulation than when positive lineup decisions were exchanged with an unidentified lineup member (choice exchanges). This is in line with earlier research (Johansson et al., 2005; Sagana et al., 2015). These findings are reasonable given that between-category changes, such as choice reversals, require a greater attentional shift than within-category changes, such as choice exchanges (Lyyra et al., 2014). An alternative but compatible explanation is that, compared with choice exchanges, choice reversals caused greater discrepancies in participants’ feeling of familiarity and were thus easier to be detected (Sauerland et al., 2015). However, the reversal versus exchange effect was limited to overall detection rates and its effect size was relatively small compared with the earlier investigation. The longer retention intervals of this study (48 h), compared with that of the earlier field study (minutes; Sagana et al., 2015), might have blurred the distinction between the choice and its outcome. Specifically, participants might have felt less confidence in their own memory and therefore refrained from vocalizing their suspicion.

As to the role of confidence in detection rates, we found that low post-identification confidence was not a predictor of whether a manipulation would be detected or endorsed (but see Sagana et al., 2013). However, the mere presence of a manipulation was sufficient to lower post-manipulation confidence. The finding carries practical and theoretical implications because it suggests that by the time witnesses are asked to appear at court they would have experienced confidence deflation and would run the risk of being perceived as unreliable. This development could be seen as positive considering that, in effect, the risk of a wrongful conviction is reduced when the identification decision is dismissed as unreliable. From that point of view, the drop in confidence can be seen as a marker of procedural errors. From a theoretical stance, it may indicate that participants experience the discrepancy between the original choice and the manipulated outcome at a metacognitive level. This fits well within the eyewitness literature about the role of confidence as a postdictor of identification accuracy (e.g., Sporer et al., 1995; Brewer and Wells, 2006; Wixted and Wells, 2017). Yet, this dissonant feeling may not be strong enough for participants to raise explicit concerns about the outcome of their decision. This hypothesis remains to be empirically tested. Additionally, the drop in confidence following the manipulation illustrates how procedural errors can obstruct justice, as a potentially accurate (original) identification decision would be dismissed. This finding underscores the importance of recording confidence at the time of the identification decision and before any sort of feedback is given to the witness (e.g., Bradfield et al., 2002; Wells et al., 2003; Charman and Wells, 2012).

To conclude, the present study suggests that although warnings are not sufficient to reduce choice blindness for identification decisions, they can provide a time-related advantage. When pre-warned, participants detect the manipulations sooner than when they were post-warned or not warned. These findings are of practical relevance as they suggest that the warning procedure can help detect mistakes in the identification procedure at an earlier stage, before it can contaminate other pieces of evidence. However, the fact that the warnings were ineffective in increasing the overall detection rates suggest that choice blindness is deeply rooted in cognition and requires more elaborate interventions (Sagana and Sauerland, 2017).

## Ethics Statement

All procedures performed in studies involving human participants were in accordance with the ethical standards of the institutional and/or national research committee and with the 1964 Helsinki declaration and its later amendments or comparable ethical standards.

## Author Contributions

AS generated the idea for the study, designed the experiment, conducted the data analysis, interpreted the results, and led the writing of this manuscript. MS contributed to the research idea and designed the experiment, helped with interpreting the results, and aided in writing up this manuscript. HM aided in interpreting the results, provided critical feedback, and contributed to writing up the manuscript.

## Conflict of Interest Statement

The authors declare that the research was conducted in the absence of any commercial or financial relationships that could be construed as a potential conflict of interest.
